# Self-Determination and Physical Exercise Adherence in the Contexts of Fitness Academies and Personal Training

**DOI:** 10.1515/hukin-2015-0052

**Published:** 2015-07-10

**Authors:** Ingi Petitemberte Klain, Dihogo Gama de Matos, José Carlos Leitão, Luís Cid, João Moutão

**Affiliations:** 1 University of Trás-os-Montes and Alto Douro (UTAD), Research Centre in Sport, Health and Human Development (CIDESD), Vila Real, Portugal.; 2 Sport Sciences School of Rio Maior (ESDRM-IPS), Research Centre in Sport, Health and Human Development (CIDESD), Vila Real, Portugal.

**Keywords:** motivation, self-determination theory, exercise adherence

## Abstract

This research aimed to analyze the validity of the relations hypothesized by the theory of self-determination in predicting adherence to physical exercise in fitness academy users and subjects following personal training. A total of 588 persons from Pelotas / RS / Brazil (405 gym users and 183 subjects following personal training) completed the Portuguese version of the three questionnaires, i.e. the Perceived Autonomy Support Climate Exercise Questionnaire, Basic Psychological Needs in the Exercise Scale and Behavioral Regulation in the Exercise Questionnaire −2. The results support the factorial structure of the questionnaires used in this sample. There was a significant multivariate effect of context on self-determination for physical exercise training [Wilks’ λ = 0.934, F (10, 576.000) = 4.03, p < 0.001, η^2^ = 0.01]. The hypothesized structural equation model, which considered the self-determination theory, showed a good fit to the data (S-B χ^2^ = 234.703; p= .001; df = 52; χ^2^/df = 4.514; SRMS = .049; NNFI = .906; CFI = .926; RMSEA = .077; RMSEA 90% CI = .067 − .088). However, in the comparative analysis, the perception of autonomy support, relatedness and competence were significantly higher in the context of personal training, while the amotivation and external regulation were significantly higher in the context of fitness academies.

## Introduction

According to the Self-Determination Theory (SDT; Deci and Ryan, 1985), the person’s motivation is not directly related to the factors of social involvement, since the influence of these factors (e.g., environment during activities, behavior of the instructors) is mediated by the satisfaction of three “key nutrients”, which include the innate basic psychological needs (BPN) of autonomy (the need to regulate their own actions), competence (the need to be effective in their actions) and relatedness (the need to search and develop connections and interpersonal relations). These basic psychological needs will determine the regulation of the person’s behavior through a *continuum* of self-determination that describes the concept of internalizing behavior, which can range from amotivation (the lack of motivation or intention to act – a lower level of self-determination of the *continuum*), through the extrinsic motivation (introjected, identified and integrated external regulation) to intrinsic motivation (the completion of the behavior due to the enjoyment and fun it provides – a higher level of self-determination of the continuum) (Deci and Ryan, 2008).

According to Deci and Ryan (2008), the central differentiation of the SDT is between autonomous regulation (that incorporates the intrinsic, integrated and identified motivation) and controlled regulation (which incorporates introjected and external motivation). In the first case, when people are autonomously motivated, they experience pleasure (guiding their behavior by decision and self-will) or feelings of self-approval of their actions. In the second case, when people are motivated in a controlled manner, they experience pressure situations which make them think, feel or behave in a particular way (i.e., manage their behavior according to external resolutions or internal pressures). Both autonomic and controlled regulations direct and influence the behavior of the subject, contrary to what happens with amotivation which reveals a lack of a regulatory process.

Applying the SDT to the context of physical exercise, the autonomy support provided by teachers can positively influence the satisfaction of basic psychological needs (BPN) of the practitioners (i.e., autonomy, competence, and relatedness), which in turn could have a positive impact on autonomous behavioral regulation. This can positively influence the welfare and behavior of individuals as well as vitality perception and adherence to physical exercise, as found in some recent studies (Moutão et al., 2012a; Vlachopoulos and Karavani, 2009) including the most recent systematic review on the application of the SDT to physical exercise (Teixeira et al., 2012), where authors conclude that a motivational profile marked by high autonomous motivation is important to sustain exercise behaviours over time, with a trend towards identified regulation predicting initial/short-term adoption more strongly than intrinsic motivation, and intrinsic motivation being more predictive of long-term exercise adherence.

However, this theoretical model of causal relations is yet to be tested in a sample of exercisers in the context of fitness academies and personal training in Brazil, in spite of being understood that the SDT can facilitate a better understanding of the factors determining the exercise adherence, and consequently allow a more effective professional intervention. Thus, the aim of this study was to analyze the validity of the causal model hypothesized by the SDT in predicting exercise adherence in fitness academy users and subjects following personal training.

## Material and Methods

### Participants

The study included a total of 588 subjects: 405 who performed physical exercise in fitness academies, including both females (n = 240, 59%) and males (n = 165, 41%) aged between 18 and 81 years (M = 35, SD = 17); and 183 who exercised in the context of personal training, also including both females (n = 142, 78%) and males (n = 41, 22%) aged between 18 and 88 years (M = 43, SD = 16). All subjects originated from Pelotas / RS / Brazil and met the following inclusion criteria: they regularly attended classes (i.e., at least two training sessions per week) in fitness academies or following personal training programs; and they signed a consent form. This research is characterized as a field work and should be considered as a mostly quantitative descriptive cross sectional study, using an intentional non-probabilistic sample.

### Measures

*Perceived Autonomy Support Climate Exercise Questionnaire* – a Portuguese version (PASECQp): is a self-report instrument adapted to the context of physical exercise by Edmunds et al. (2006), based on the original version of *Perceived Autonomy Support: Health Care Climate Questionnaire* (Williams et al., 1996), translated and validated for the Portuguese language (Moutão et al., 2012c). This questionnaire consists of six items, which contribute to a single factor that evaluates the perception of autonomy support given by the physical educator (e.g. demonstrates confidence in my ability to perform the exercises). The answer is given on a Likert scale of 1–7, corresponding to “Strongly Disagree” option to the value 1 and “Strongly Agree” to the value 7.

Basic Psychological Needs in the Exercise Scale – a Portuguese (BPNESp) version: is a self-report instrument developed specifically for the context of physical exercise by Vlachopoulos and Michailidou (2006), subsequently translated and validated for the Portuguese language (Moutão et al., 2012d); it is used to evaluate the perception that people have of the level of satisfaction of their BPN. This questionnaire consists of 12 items distributed between the autonomy factors (e.g., I exercise according to what I intend to do), competence (e.g., I feel that physical exercise is an activity that I do very well) and relatedness (e.g., I have a close relationship with the people with whom I exercise). The answers are given on a Likert scale of 1–5, corresponding to the “Strongly Disagree” option to the value 1 and “Strongly Agree” to the value 5.

Behavioral regulation in the exercise questionnaire 2 – a Portuguese (BREQp-2) version: is an instrument of self-report developed by Markland and Tobin (2004), translated and validated for the Portuguese language (Palmeira et al., 2007) and later validated in the context of physical exercise in fitness academies (Cid et al., 2012). This questionnaire allows to evaluate the type of motivational regulation related to physical exercise and consists of 19 items that assess amotivation and extrinsic behavioral adjustments, introjected, identified and intrinsic (e.g., I like my workouts, I exercise because it is fun). The answers are given on a Likert scale of 0–4, corresponding to the “Strongly Disagree” option to the value 0 and the other end “Agree” to the value 4. Adherence to exercise was assessed by self-report and analysed as “persistence”, meaning the duration of time (months) from initiation to discontinuation of the exercise program. This classification was defined through a survey that inquired how long an individual was practicing physical exercise in the context that he/she currently attended to.

### Procedures

Firstly, the owners and/or directors of the academies and/or customized training centers were contacted via formal invitation and submission of the pre-research project, in order to get the required approval for conducting this research at each site. Fitness academies were chosen for their convenience and in the institutions where permission was granted, the participants were approached before initiating their exercise session. Since the participation in the survey was voluntary, not all academy users answered the questionnaires.

All applied methodological procedures were approved by the Ethics Committee on Human Research of the School of Physical Education, Federal University of Pelotas, under the license number of 016/2012. Furthermore, data was only collected after the volunteers were rightfully informed and the consent form signed, authorizing their participation in the investigation and agreeing with the publication of the results, keeping their personal data anonymous.

The procedures followed the rules of ethics in human research established in the Resolution No. 251 of 07/08/1997 of the National Board of Health and in the Resolution No. 196 of 10/10/1996 that features the regulatory guidelines for research involving human subjects, in accordance with the ethical principles of the Declaration of Helsinki, by the “World Medical Association” (WMA, 2008).

### Statistical analysis

Since this is the first time the instruments used here were applied to a group of Brazilian subjects, the construct validity of all measurement models used was examined by confirmatory factor analysis (CFA). In addition, values of internal consistency (Cronbach’s alpha) for each factor were also calculated using a cutoff value of .70, as suggested by Nunnally (1978), for a reasonable internal consistency of each factor.

To perform the CFA and for assessing the adequacy of the outlined structural equation model, the estimation method of maximum likelihood was used (ML: *MaximumLikelihood*) which assesses the statistical model by chi-square (χ^2^ *Chi-Square*). Considering that the theory underlying the method of ML estimation assumes multi-normality of items (Kahn, 2006), the Mardia’s Test (Mardia, 1970) is required to evaluate this assumption. Since the normalized Mardia value for our sample exceeded 5 (Byrne, 2009) in most of the analyzed models, a robust method was used to correct the χ^2^ values for non-normality of the data distribution (Chou and Bentler, 1995). Therefore, the value of Satorra-Bentler χ^2^ was presented (S-B χ^2^: see Satorra and Bentler, 1994). In addition to the test S-B χ^2^, the respective degrees of freedom (df), the level of significance (p) and robust estimates of indexes of fit more consensual in the literature (Hair et al., 2009) were also presented, i.e.: *Standardized Root Mean Square Residual* (SRMR), the *Comparative Fit Index* (CFI), the *Non-normed Fit Index* (NNFI), *Root Mean Square Error of Approximation (*RMSEA) and the respective 90% confidence interval (CI). For these indexes, cutoff values suggested by Hu and Bentler (1999) were adopted: SRMR ≤ .080, CFI and NNFI ≥ .950 and RMSEA ≤ .060. Software EQSWIN (version 6.1) was used to perform the statistical analyses.

Regarding subsequent univariate statistical analysis, techniques of descriptive statistics (mean, standard deviation) were used to determine normality (asymmetry, kurtosis) and correlation between variables was assessed using the Pearson product-moment correlation coefficient (Pearson’s r). To analyze the effect of the context of practice (i.e. personal training vs fitness academies) on the dependent variables of the SDT, the multivariate statistical technique MANOVA was used. The independent effect size attributed to the variable was estimated by calculating the Eta squared (*η^2^*) and interpreted according to the cutoff values proposed by Ellis (2010): small effects for *η^2^* ≥ 0.01; average effects for *η^2^* ≥ 0.06; and large effects *η^2^* ≥ 0.14. These calculations were performed with SPSS statistical software (version 20.0), and the level of significance was set at p <.05.

## Results

### Adjustment of measurement models

Results show that the obtained CFA models present acceptable adjustment indexes. However, with regard to the questionnaire BREQ-2p it was only possible to adjust a model after eliminating the item 17. Problems with this item had already been identified in previous works when validating this questionnaire in the context of physical exercise in fitness academies (Cid et al., 2012). In the present study, there was also a low weight factor (WF = .25) of the item 4 (intrinsic regulation), thus it was also removed from the model. The factor weights varied between .68 and .78 in the PASECQp, between .38 and .80 in BPNESp and between .37 and .69 in the BREQ-2p (only the item 14 - identified regulation had a weight below .50). These results, as well as the values of internal validity presented in Table 3 for each scale, demonstrate the construct validity of the Portuguese versions of the PASECQp, BPNESp and the BREQp-2 in Brazil, for this group of participants in particular, and ensure the quality of the data, allowing its use in the subsequent statistical analysis.

### Descriptive analysis of data

In Table 2 the values of descriptive statistics and dispersion measures of the latent variables are presented taking into account all participants in this study.

#### Internal consistency

Regarding the internal consistency, all scales had Cronbach’s alpha values above the defined cutoff level (i.e. .70).

### Correlational analysis

The correlation matrix shows a relation between the variables that is consistent with the TAD and thus, the autonomy support is positively related with the satisfaction of the three BPN. Furthermore, the three BPN are negatively related to amotivation and the most controlling forms of behavior regulation (i.e. introjected and external) and positively related to the more autonomous forms of regulation (i.e. identified and intrinsic). It also appears that there is a negative relationship between the motivational forms at the opposite ends of the self-determination continuum (i.e. intrinsic and external) and stronger relations between the closest forms of regulation (e.g., intrinsic and identified; introjected and external). Finally, with regard to exercise adherence, the less self-determined forms are negatively related with persistence and the more self-determined regulations are positively related with persistence.

### Predictive effect of SDT on adherence to exercise

Regarding the aims of this study, we analyzed the causal relationships proposed by the SDT and their effect on adherence to exercise using a structural equation model. For the sake of model simplification, an overall index of satisfaction of basic psychological needs was used, consisting of the three psychological needs, which were validated in a measurement model as a 2nd order factor (Moutão et al., 2012d). The use of indexes of this nature is not unheard of and had been empirically supported in several studies (Deci et al., 2001; Gagné, 2003; Ntoumanis, 2005; Vlachopoulos, 2007). For the same reason, a measure of autonomic regulation was used, which consisted of the arithmetic mean between the two regulation scales of higher self-determination (i.e. identified regulation and intrinsic regulation). This measure of autonomic regulation is consistent with the theoretical assumptions of the SDT (Deci and Ryan, 1985, 2002) and has recently been psychometrically validated in Portuguese subjects who perform physical exercise (Cid et al., 2012). In the present study, these indexes had acceptable levels of internal consistency, mainly autonomous motivation (.68), controlled motivation (.67) and basic psychological needs (.83).

Results also showed that the model had a good fit to the sample data (S-B *χ^2^* = 234.703; *p*=.001;df = 52; *χ^2^*/df = 4.514; SRMS = .049; NNFI = .906;CFI = .926; RMSEA = .077; RMSEA 90% CI = .067 – .088).

The squared multiple correlations indicate that supporting autonomy explains 40% of the satisfaction of BPN and the latter explains 32% of autonomic regulation. In addition, autonomic regulation explains 6% of the variation in adherence to exercise.

### Comparative analysis

According to defined methodology, the effect of the practice context (personal training vs fitness academy) on the latent variables was analyzed using a MANOVA. Results revealed the existence of a significant multivariate effect of the context on the motives to practice physical exercise [Wilks’ λ = 0.934, F (10, 576.000) = 4.03, p < 0.001, η^2^ = 0.01]. Even considering that this effect sized could be statistically small (η^2^ = 0.01), it is important to note that any positive effect has practical importance since the drop-out rate for those engaged in newly established exercise regimens is 40–65% in the first 3–6 months (Annesi, 2003).

Considering this effect, in Table 4 we present the mean values obtained in each of the latent variables (taking into account the context of practice), along with the respective analysis of variance statistics (F), the level of significance (p) and effect size (η^2^).

It is possible to verify that subjects undergoing personal training perceive significantly greater autonomy support by the instructor, which seems to be reflected in a significantly higher perception of satisfaction in their psychological need for relations. Consistent with these results, fitness academy users differ by having significantly higher levels of amotivation and external regulation, which is reflected in a significantly lower adherence to exercise.

## Discussion

This work aimed to study the influence of autonomy support, induced by the physical educator, on the satisfaction of BPN and motivational regulation in individuals attending fitness academies as well as following personal training and its impact on adherence to exercise.

Since there was no previous information, trust values and construct validity for the questionnaires PASECQp, BPNESp and BREQp-2, for individuals practicing physical exercise in Brazil, were first determined. The results obtained with the collected sample showed the reliability and construct validity of these questionnaires, ensuring the quality of data obtained.

Still, with regard to the BREQp-2, the measurement model only fitted the data collected after the elimination of the item 17. Problems with this item had already been observed, both in the original study (Markland and Tobin, 2004) and in a recent study on Portuguese subjects who performed physical exercise (Cid et al., 2012). In addition, there was also the need to delete the item 4 due to its weak association with the factor it should be associated with (i.e. intrinsic regulation). This emphasizes the need for a semantic revision in future studies. Nevertheless, the elimination of these items in the calculation of intrinsic and identified regulation contributed to obtain a more realistic value in assessing the constructs of this scale. It also emphasized the importance of pre-testing the measurement models of the questionnaires used, which is even more relevant when there is no previous information. Regarding the internal consistency (i.e., *Cronbach’ s alpha*), all scales used had alpha values above the determined cutoff level (i.e., 0.70), revealing that their items contributed in a satisfactory way to measure the same factor (i.e. the psychological attribute).

The correlation analysis between the variables revealed a pattern consistent with the theoretical assumptions of the SDT relations (Deci and Ryan, 1985), since different types of motivational regulation were positively correlated with the closest constructs (e.g., identified and intrinsic or external and introjected) and negatively with the more distant (e.g., external and intrinsic). This confirms the “simplex” correlation model mentioned by Ryan (1995), which indicates that the *Continuum* variables are ordered according to their conceptual similarity. Likewise, regarding the adherence to exercise, the less self-determined forms correlate negatively with persistence and the more self-determined regulations correlate positively with persistence, which is consistent with some recent studies on this issue (Moutão et al., 2012a.).

Regarding the adjustment of a structural equation model based on causal relationships suggested by the SDT, results confirmed that the autonomy support offered by the physical educator and perceived by the individual practicing physical exercise would promote the satisfaction of their BPN (*β*= .64; p = < .001). This had a positive impact (*β*= .55; p = < .001) on the regulation of their behavior towards more autonomous forms, which would ultimately have a positive impact (*β*= .25; *p* = < .001) on adherence to exercise. According to the study’s results, autonomy support given by the physical educator (i.e., offers choices, minimizing pressure and control, etc.), favored the satisfaction of basic psychological needs and consists of the basis of self-determined behavior for practicing physical exercise. This effect of a motivational environment induced by the instructors on the individuals’ motivation was mediated by the satisfaction of basic psychological needs, as suggested theoretically by Ryan and Deci (2007) and empirically by Moutão et al. (2012a).

Considering the comparative analysis between contexts, we found that individuals undergoing personal training feel greater autonomy support given by the physical educator, which is reflected in a significantly higher perception of satisfaction of their psychological need of relation. This fact may occur due to the closest monitoring by the instructor. According to the SDT, humans are inherently motivated to feel affiliated to others within their social reality (i.e., relatedness). Consistent with these results, individuals from fitness academies differ by having significantly higher levels of amotivation and external regulation. Although amotivation theoretically reflects the fact that the individual does not perform the behavior, nor intends to do so, it may also be present in individuals already engaged in an activity, occurring when the subject does not value (or fail to cherish) the activity, not feeling (or failing to feel) competent to execute it, and/or does not believe (or cannot believe) in its results (Ryan and Deci, 2007). With regard to external regulation, the individual performs the behavior to obtain rewards or avoid punishments. External regulation corresponds to the less self-determined type of extrinsic motivation and is the most basic and least autonomous extrinsic motivation, in which externally regulated behaviors are performed so that the individual obtains a reward or satisfies an external demand (Reeve et al., 2004).

Results show that individuals in the context of personal training have more self-determined forms of regulation and adhere more to physical exercise. These findings are in accordance with the assumptions of the SDT, suggesting that more self-determined people are more likely to compromise with certain behaviors than those with low self-determination (Deci and Ryan, 2000). Current research found that subjects with higher self-determination to practice physical exercise and sports had higher adherence to their activities (Edmunds et al., 2006; Ntoumanis, 2005; Wilson and Rodgers, 2004). In conclusion, we believe that the SDT can help in a better understanding of the quality of motivation that leads fitness academy users and subjects following personal training to adhere (or not) to physical exercise and that it can enable more effective professional involvement.

Some limitations of the current study are that it used self-report questionnaires (i.e., it is subject to errors of interpretation), has a cross-sectional design and was developed in a specific setting and population limiting the generalization of the results to other contexts. In spite of these limitations, this study provides novel evidence of how the Self-Determination Theory could be used for promoting exercise adherence by identifying some of the moderators and mediators responsible for this behaviour allowing the future replications of these results throughout the development of intervention based on longitudinal studies and objective measures.

## Figures and Tables

**Figure 1:**
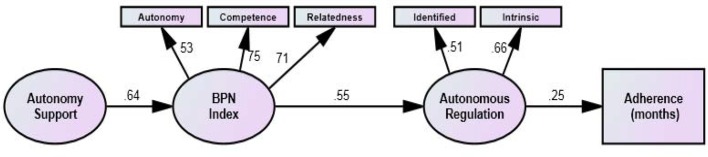
Structural equation model analyzing the causal relations between the variables of SDT and its effect on adherence to exercise. BPN = basic psychological need. All parameters are presented in a standardized manner and represent significant effects (p <.01)

**Table 1:** Confirmatory factor analysis and related indexes of fit obtained for each of the questionnaires used

Models	*S-B*χ^2^	p	*df*	χ^2^/*df*	SRMS	NNFI	CFI	RMSEA	90% IC
PASECQp	63.969	.000	9	7.108	.034	.945	.967	.102	.079 – .126
BPNESp	212.956	.000	51	4.176	.068	.907	.928	.074	.063 – .084
BREQ-2p^*^	281.588	.000	125	2.583	.051	.896	.916	.052	.045 – .059

*S-B*χ*^2^ – Chi-square with correction Satorra-Bentler; p = degree of significance of the chi-square test; df = Degrees of freedom; χ^2^/df = ratio of chi square to its degrees of freedom*; *SRMS = Standardized Root Mean Square; NNFI = Bentler-Bonnett Non normed Fit Index; CFI = Comparative Fit Index; RMSEA = Root Mean-Squared Error of Approximation; 90% IC = confidence interval 90% to RMSEA.*

* After elimination of items 4 (intrinsic regulation) and 17 (identified regulation).

**Table 2:** Descriptive statistics, measures of central tendency and dispersion, and the amount of internal reliability of latent variables

Latent variable	*M*	*SD*	Min-Max	Asymmetry	Flattening	***α***
1. Support Autonomy	5.50	1.07	1.00 – 7.00	−0.421	0.158	.88
2. BPN Autonomy	3.83	0.71	1.75 – 5.00	−0.359	−0.014	.67
3. BPN Competence	4.12	0.59	2.00 – 5.00	−0.378	0.251	.76
4. BPN Relationship	4.15	0.67	1.00 – 5.00	−0.772	1.287	.82
5. Amotivation	0.27	0.59	0.00 – 4.00	2.923	9.868	.65
6. External Regulation	0.40	0.72	0.00 – 4.00	2.197	4.999	.74
7. Introjected Regulation	1.66	1.14	0.00 – 5.00	0.422	−0.555	.66
8. Regulation Identified	3.16	0.70	0.50 – 5.00	−0.817	0.622	.52
9. Intrinsic Regulation	3.05	0.75	0.50 – 5.00	−0.793	0.348	.67
10. Persistence (months)	21.01	26.13	1 – 180	2.230	6.203	-

M = mean; SD = standard deviation; Min-Max = value minimum and maximum; α = Cronbach’s alpha; BPN = basic psychological need.

**Table 3:** Matrix of correlations of the latent variables

Latent Variable	1.	2.	3.	4.	5.	6.	7.	8.	9.	10.
1. Support Autonomy	-									
2. BPN Autonomy	.400^**^	-								
3. BPN Competence	.405^**^	.406^**^	-							
4. BPN Relationship	.431^**^	.322^**^	.542^**^	-						
5. Amotivation	−.086^*^	.024	−.124^**^	−.074	-					
6. External Regulation	−.080	−.012	−.104^*^	−.032	.504^**^	-				
7. Introjected Regulation	.111^**^	.089^*^	.163^**^	.185^**^	.093^*^	.197^**^	-			
8. Regulation Identified	.177^**^	.116^**^	.339^**^	.231^**^	−.094^*^	.012	.416^**^	-		
9. Intrinsic Regulation	.213^**^	.196^**^	.292^**^	.270^**^	−.121^**^	−.120^**^	.147^**^	.402^**^	-	
10. Persistence	.031	.103^*^	.084^*^	.078	−.116^**^	−.124^**^	.021	.174^**^	.130^**^	-

Min-Max = value minimum and maximum; M = mean; SD = standard deviation; BPN = basic psychological need.

** p< .01;

* p< .05

**Table 4:** Comparative analysis of motivational regulations for physical exercise considering the contexts of fitness academy and personal training

	Fitness Academy			Personal training			*F*	*p*	*η^2^*

	Mean	SD	Min-Max	Mean	SD	Min-Max			
Support Autonomy^*^	5.44	1.07	1.00–7.00	5.63	1.07	2.00–7.00	3.873	.050	.007
BPN Autonomy	3.84	0.72	1.75–5.00	3.83	0.68	2.00–5.00	0.014	.907	.000
BPN Competence	4.13	0.60	2.00–5.00	4.10	0.56	2.00–5.00	0.219	.640	.000
BPN Relationship^**^	4.09	0.69	1.00–5.00	4.29	0.61	1.75–5.00	11.193	.001	.019
Amotivation^**^	0.32	0.64	0.00–4.00	0.18	0.46	0.00–3.50	6.246	.013	.011
External Regulation^*^	0.45	0.78	0.00–4.00	0.30	0.53	0.00–2.50	5.999	.015	.010
Introjected Regulation	1.63	1.17	0.00–5.00	1.71	1.07	0.00–4.00	0.638	.425	.001
Regulation Identified	3.13	0.74	0.50–5.00	3.61	0.59	1.00–4.00	1.760	.185	.003
Intrinsic Regulation	3.00	0.77	0.50–5.00	3.08	0.71	0.50–4.00	0.515	.473	.001
Persistence (months)	18.63	23.92	1–180	26.28	29.87	1–168	10.831	.001	.018

*Min-Max = value minimum and maximum; M = mean; F =* Value statistical test; *η^2^* = eta squared; *BPN = basic psychological need.*

* = *p*<.05;

** = *p*<.01.
